# Effects of COVID-19 Pandemic Confinement in Patients With Cognitive Impairment

**DOI:** 10.3389/fneur.2020.589901

**Published:** 2020-11-24

**Authors:** Ainara Barguilla, Aida Fernández-Lebrero, Isabel Estragués-Gázquez, Greta García-Escobar, Irene Navalpotro-Gómez, Rosa María Manero, Víctor Puente-Periz, Jaume Roquer, Albert Puig-Pijoan

**Affiliations:** ^1^Neurology Department, Hospital del Mar, Barcelona, Spain; ^2^Cognitive Impairment and Movement Disorders Unit, Neurology Department, Hospital del Mar, Barcelona, Spain; ^3^Neurofunctionality and Language Group, Neurosciences Program, Hospital del Mar Research Institute (IMIM), Barcelona, Spain

**Keywords:** COVID- 19, SARS - CoV-2, dementia, depression, cognitive impairment, anxiety, neuropsychiatric

## Abstract

**Introduction:** State of emergency caused by COVID-19 pandemic and subsequent lockdown hit Spain on 14th March 2020 and lasted until 21st June 2020. Social isolation measures were applied. Medical attention was focused on COVID-19. Primary and social care were mainly performed by telephone. This exceptional situation may affect especially vulnerable patients such as people living with dementia. Our aim was to describe the influence of restrictive measures on patients living with mild cognitive decline and dementia evaluating SARS-CoV2 infection, changes in routines, cognitive decline stage, neuropsychiatric symptoms, delirium, falls, caregiver stress, and access to sanitary care.

**Materials and Methods:** We gathered MCI and dementia patients with clinical follow-up before and after confinement from DegMar registry (Hospital del Mar). A telephone *ad-hoc* questionnaire was administered. Global status was assessed using CDR scale. Changes in neuropsychiatric symptoms were assessed by Neuropsychiatric Inventory (NPI) and retrospective interview for pre-confinement base characteristics.

**Results:** We contacted a total of 60 patients, age 75.4 years ± 5,192. 53.3% were women. Alzheimer's Disease (41.7%) and Mild Cognitive Impairment (25%) were the most prevalent diagnosis. Remaining cases included different dementia disorders. A total of 10% of patients had been diagnosed with SARS-CoV-2. During confinement 70% of patients abandoned previous daily activities, 60% had cognitive worsening reported by relatives/caretakers, 15% presented delirium episodes, and 13% suffered increased incidence of falls. Caregivers reported an increased burden in 41% cases and burnout in 11% cases. 16% reported difficulties accessing medical care, 33% received medical phone assistance, 20% needed emergency care and 21% had changes in psychopharmacological therapies. Neuropsychiatric profile globally worsened (*p* < 0.000), also in particular items like agitation (*p* = 0.003), depression (*p* < 0.000), anxiety (*p* < 0.000) and changes in appetite (*p* = 0.004).

**Conclusion:** SARS-CoV2-related lockdown resulted in an important effect over social and cognitive spheres and worsening of neuropsychiatric traits in patients living with mild cognitive decline and dementia. Although the uncertainty regarding the evolution of the pandemic makes strategy difficult, we need to reach patients and caregivers and develop adequate strategies to reinforce and adapt social and health care.

## Introduction

The current coronavirus disease global pandemic, caused by SARS-CoV2 (Severe Acute Respiratory Syndrome Coronavirus 2), was first acknowledged in Wuhan in December 2019 (1). From then on, its high infectivity and severity collapsed the health systems of numerous countries and forced preventive measures such as social distancing and lockdown throughout the world. The World Health Organization declared the COVID-19 pandemic on 11 March 2020 (2). Measures varied hugely between countries and while social distancing has been generally the norm, there has not been a worldwide standard response.

State of emergency and subsequent lockdown started in Spain on 14 March 2020 and, through gradual de-escalation, lasted until 21st of June 2020. During the lockdown, the population was prevented from outdoor exercising and maintaining contact with friends and family. Though taking care of dependent relatives (shopping for them, medication management.) was an exception to circulation restrictions, family contact was highly discouraged. Medical attention during the state of emergency was focused on COVID-19 and primary and social care were mainly substituted by telephone visits when possible. Day centers and cognitive stimulation centers, along with outpatient care, were suspended. Only critical attention was guaranteed and even after the state of emergency ended, patient care changed: on-site interviews were reduced and telephonic attention encouraged.

Social impact of the pandemic has been huge with rising levels of poverty and unemployment affecting the care of the most vulnerable (3). Among all the population affected by the pandemic, elderly people have been in the highest risk of mortality, so far, most deaths have been over the age of 70 (4, 5).

More than 50 million people worldwide live with dementia (6). Prevalence of mild cognitive impairment in older of 60 years ranges between 16 and 20%, half of whom will develop dementia throughout their lives (7, 8). In Spain, 80% of people with dementia live at home and depend on their family as caregivers (8).

Patients living with neurodegenerative diseases are especially vulnerable to infections and changes in their routines. Social isolation has been associated with negative outcomes (9). COVID-19 pandemic and previously described restrictive measures such as social distancing may lead, thus, to a worsening of their cognitive status, functional performance, mood, behavior, and sleep (10, 11). Other complications such as falls and delirium might be favored by the lack of usual care. The difficulty to access the health care system, often magnified by the lack of an easy to use telematic platform for the elderly, may increase the anxiety and feeling of being abandoned both in patients and, very importantly, in caregivers (12).

Our aim was to make the effects of restrictive measures on patients living with dementia and cognitive impairment visible, focusing on changes in their daily routines, global cognitive/functional status, delirium, falls, caregiver stress, neuropsychiatric symptoms, and also their perception of the limitation to access to sanitary resources.

Describing the impact of this new environment over our patients is the first step toward adapting our assistance to their needs and designing new strategies in order to improve their care and therefore their quality of life.

## Materials and Methods

### Subjects

In our cognitive impairment and movement disorders unit we gather patient data in the DegMar registry. Patients participating in the DegMar project sign informed consent approved by the local ethics committee (2018/7805/I) which implies the use of medical information for research purposes. Patients recruited for CogVid study and their families were asked for specific permission in order to include their clinical data in this study.

We gathered patients from DegMar registry with previous follow up within 6 months before the state of alarm in order to have the most updated previous cognitive and functional status. We excluded healthy individuals and those with subjective cognitive decline. We also excluded patients with previous comorbid psychiatric disorders or suffering from mourning deriving from family loss. From these patients, we interviewed the ones with previously programmed telephonic follow-up as a part of our daily clinical practice.

The clinical diagnosis of the subjects was stated according to clinical history. Severity of the cognitive impairment was staged by the Clinical Dementia Rating Scale (CDR) global score (13).

### Assessment

We created a telephone *ad-hoc* questionnaire “CogVid Hospital del Mar questionnaire” to measure functional and neuropsychiatric changes experienced by patients and caregivers of this sample during the period of confinement (from March to May 2020). We chose an *ad-hoc* non-standarized, non-validated questionnaire in order to gather as much information as soon as possible: immediacy in our case made the information more reliable. It made a useful tool for guiding the interviewers and gather information in an homogenous way. The questionnaire included comprehension/adaptation to confinement and protective measures, change of residence, social services support loss, primary care attention, and psychopharmacological treatment adjustment during confinement period. We also registered infection symptoms, falls, interruption in cognitive stimulation programs, and loss of day to day routines (shopping, strolling…). Caregiver stress was also assessed. We registered access to institutions and professional societies' online resources and if any psychological support or relief activities (mindfulness, physical activity) were practiced. The interview was conducted with the caregiver, family, or live-in resident of the patient.

This telephone questionnaire was conducted from June to July during the programmed medical consultation, by telephone, and lasted about 20 min.

A model of the used questionnaire (CogVid Hospital del Mar Questionnaire) is attached in Annex 1.

In order to describe possible changes in the neuropsychiatric sphere, the Neuropsychiatric Inventory (NPI) was administered after lockdown altogether with the questionnaire. We also inquired about neuropsychiatric symptoms prior to state of alarm. This scale (14) is a structured caregiver-based interview that quantifies behavioral changes detected in the last 4 weeks and it consists on 12 items that match the following domains: delusions, hallucinations, agitation, dysphoria, anxiety, apathy, irritability, euphoria, disinhibition, aberrant motor behavior, nighttime behavior disturbances, and appetite/eating abnormalities. In addition, severity and frequency are graduated in each of them. For scoring, the severity scale is multiplied with the frequency scale and the score for each domain is obtained. The total score is the result of the sum of all the domains. The NPI is a widely used tool for measuring psychological disturbances in people with dementia (15).

The clinical diagnosis of the subjects was stated according to clinical history.

Severity of the cognitive impairment was staged by the Clinical Dementia Rating Scale (CDR) global score (13). CDR stage previous to lockdown was gathered from clinical history records from the last visit, 6 months before the state of alarm.

### Statistical Analysis

Descriptive data of quantitative variables from the questionnaire are shown in mean ± (SD) when normal and median (range) when not normal.

Categorical variables were summarized using frequency counts and percentages. Absolute and relative frequencies were used for qualitative variables. For comparing quantitative continuous normal variables, we used one-way ANOVA and for categorical variables we used Pearson's chi-squared test. We used Wilcoxon test to compare midrange in paired samples both normal ordinal and not normal quantitative variables. The significance threshold was set at *P* < 0.05. Statistical analysis was performed using IBM SPSS Statistics for Windows, Version 19.0 (IBM Corp., Armonk, NY, USA).

## Results

We contacted a total of 60 patients. All the patients and families gave permission for participating in the study and answered our questionnaire without difficulties.

**Figure 1:**
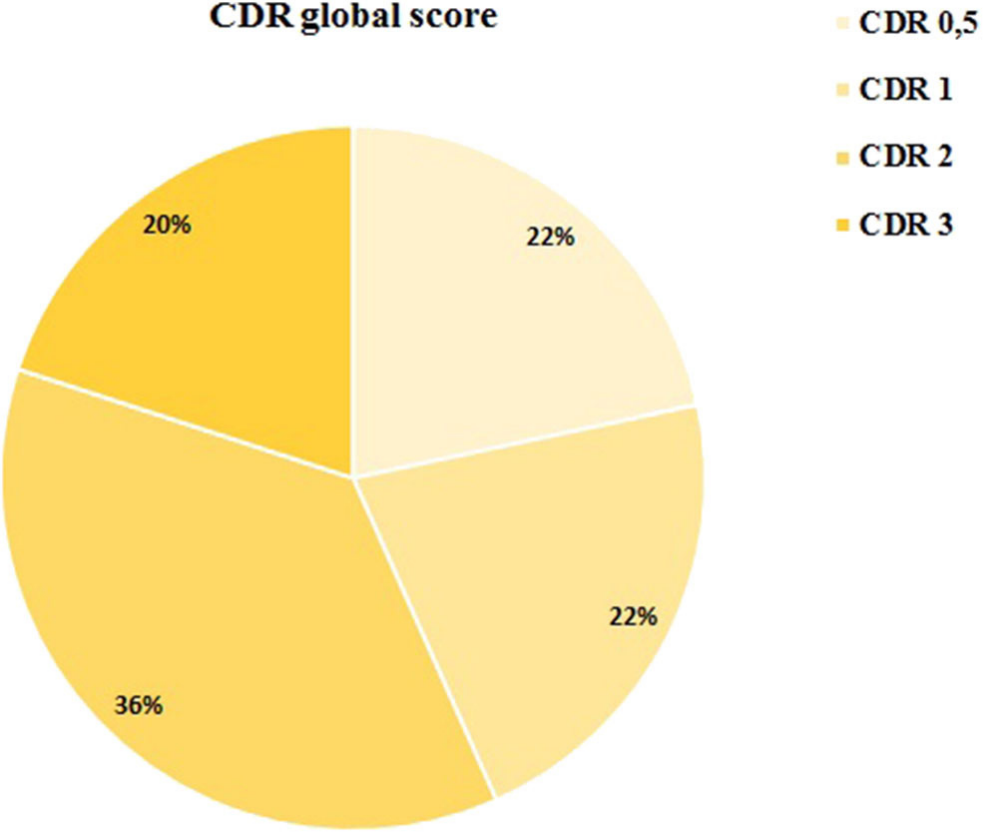
Dementia stage distribution (CDR).

Mean age was 75.4 years ± 5.2. 53.3% of the patients were women 46.7%, men. Prior to the state of alarm, 13 patients had very mild dementia (CDR 0.5), 13 mild dementia (CDR 1), 22 moderate dementia (CDR 2), and 12 severe dementia CDR 3. A graphic representation of the CDR distribution is provided in Figure 1.

When age and sex distribution in dementia severity stages was analyzed there was no significative difference between groups (Table 1).

**Table 1:** Sample characteristics according to CDR classification.

**Characteristics**	**Sample**	**CDR 0.5**	**CDR 1**	**CDR 2**	**CDR 3**	***P***
*n*. (%)	60 (100)	13 (21.7)	13 (21.7)	22 (36.7)	12 (20)	
Sex, *n* (% women)	32 (53.3)	7 (53.8)	7 (53.8)	10 (45.5)	8 (66.7)	NS^*^
Age, mean (SD)	75.4 (5.2)	77.0 (4.2)	75.5 (4.2)	75.4 (5.1)	73.58 (7.1)	NS^**^

* *Pearson's chi-squared test*,

** *one-way ANOVA*.

**Figure 2:**
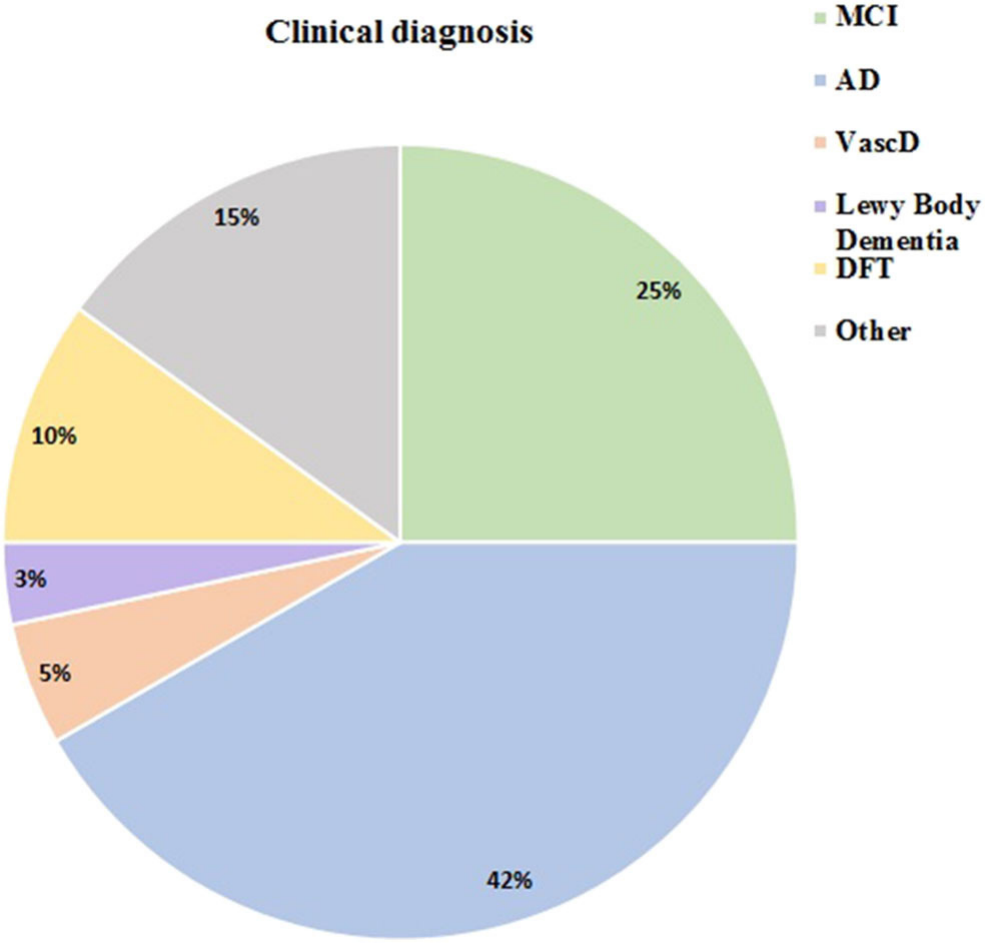
Clinical diagnosis distribution.

From all the interviewed patients, 25 (41.7%) had Alzheimer Disease (AD) diagnosis, 15 (25%) MCI, 6 patients were diagnosed with behavioral variant frontotemporal dementia (bvFTD) 3 patients were diagnosed with vascular dementia (VD), and 2 with Lewy body disease (LBD). Others including etiologically mixed dementias, Lewy Body disease, vascular dementia, and PSP accounted for the rest of the patients. A graphic representation of patient distribution by diagnosis is provided in Figure 2.

Table 2. Gathers the most important changes in our patients during the lockdown.

**Table 2:** Collected data from total sample and according to CDR classification.

		**Sample (*n* = 60)**	**CDR 0.5 (*n* = 13)**	**CDR 1 (*n* = 13)**	**CDR 2 (*n* = 22)**	**CDR 3 (*n* = 12)**
**Social changes**						
	Change of residence (1)	10 (16.7)	2 (15.4)	2 (15.4)	4 (18.2)	2 (15.4)
	Living alone (2)	12 (20.0)	4 (30.8)	5 (38.5)	1 (4.5)	2 (16.7)
	Loss of usual daily activities (3)	42 (70)	12 (92.3)	10 (76.9)	15 (68.2)	5 (41.7)
**Clinical changes**						
	Perception of cognitive worsening (4)	36 (60)	8 (61.5)	8 (61.5)	14 (63.6)	6 (50)
	Subjective mood/behavioral changes	32 (53.3)	6 (46.2)	7 (53.8)	12 (54.5)	7 (58.3)
	Mood/behavioral changes (increased NPI total score)	39 (65)	7 (53.8)	8 (61.5)	16 (72.7)	8 (66.7)
	Acute confusional state	9 (15)	0	2 (15.5)	2 (9.1)	5 (41.7)
	Increased incidence of falls	8 (13.3)	2 (15.4)	1 (7.7)	5 (22.7)	0
**Covid-19 related aspects**						
	Confirmed cases	6 (10)	0	0	1 (4.5)	5 (41.5)
	Oxygen therapy required	5 (8.3)	0	0	1 (4.5)	4 (33.3)
**Medical care**						
	Perception of difficulties in accessing care	10 (16.7)	2 (15.4)	0	4 (18.2)	4 (33.3)
	Medical phone assistance provided	20 (33.3)	3 (23.1)	4 (30.8)	10 (45.4)	3 (25)
	Standard medical consultation provided	4 (6.7)	2 (15.4)	1 (7.7)	0	1 (8.3)
	Emergency care provided	11 (18.3)	1 (7.7)	2 (15.4)	1 (4.5)	7 (58.3)
	Changes in psychopharmacological therapies	13 (21.7)	1 (7.7)	2 (15.4)	3 (13.7)	7 (58.3)
**Caregiver**						
	Perception of increased caregiver burden (5)	25 (41.7)	5 (38.5)	3 (23.1)	11 (50)	6 (50)
	Subjective caregiver burnout	7 (11.7)	1 (7.7)	0	2 (9.1)	4 (33.3)
	Use of support guidelines	2 (3.3)	0	1 (7.7)	1 (4.5)	0

*Data are shown as number (percentage) of affirmative responses*.

*(1) Change of residence: referring to institutionalization or moving in with a relative*.

*(2) Living alone: patients living on their own without continued assistance or living in relative*.

*(3) Loss of usual daily activities referring to activities asked in the questionnaire: social meetings, daycare center, cognitive stimulation, visiting relative, taking care of other family members, practicing sports or strolling, shopping, reading, watching TV*.

*(4) Perception of cognitive worsening: asked to the interviewed caretaker as a subjective question*.

*(5) Perception of increased caregiver burden: asked to the interviewed caretaker as a subjective question*.

Before lockdown, the previous social situation of our patients was: 6 institutionalized (10%), 30 (50%) independent at home and 24 (40%) supervised at home. During the state of alarm, 10 patients (17%) changed residence. No relation between functional status (CDR) and residence change was found.

Only 12 (20%) of our patients lived alone (without additional help at home or other live-in caretaker) during lockdown, 9 in the early stages of dementia (CDR 0.5–1) but also 3 with moderate-severe dementia.

During lockdown, only 6 (10%) of our patients were infected by SARS-CoV-2. All were cases of advanced stages of dementia (CDR3). Three of the patients infected were previously institutionalized, 1 (1.3%) of them died from SARS-CoV-2 infection. Three of the patients were supervised at home and remained supervised at home after the infection. Four of the SARS-CoV-2 infected patients required urgent hospitalization. Two other patients required hospitalization but for other causes.

Forty eight (70%) of our patients abandoned previous daily activities. 43.3% of our patients, the ones previously socially active, stopped attending social reunions such as daycare centers, third-age reunions, or social centers. 28.3% of our patients were previously attending cognitive stimulation in specific centers, which were closed and the activities canceled. 41.7% of our patients who previously attended gyms or went out on strolls stopped physical activity. 20% ceased to go out for daily shopping and 31% stopped visiting other family members as they did before.

In 36 (60%) patients, cognitive worsening was reported by family or caretakers, 8 among the mild cognitive decline group, 8 in the mild dementia group, and 7 in the advanced dementia group. Fourteen patients, 38% of those reported as cognitive worsening, were in dementia stage CDR2.

Functional status defined as CDR stage did not change significantly during lockdown.

When comparing prior and after lockdown CDR stages of our patients we did not find a significant difference (*p* = 0.14).

NPI score overall raised during lockdown. NPI score before lockdown was 3 (0–30). Total NPI score after lockdown was 8 (0–48). When analyzing differences between NPI before and after lockdown, we got a significant difference (*p* < 0.000).

When analyzing all the items in the NPI test, some of them revealed a significant difference before and after lockdown. These items were: agitation (*p* = 0.003), depression (*p* < 0.000), anxiety (*p* < 0.000), and changes in appetite (*p* = 0.004).

**Figure 3:**
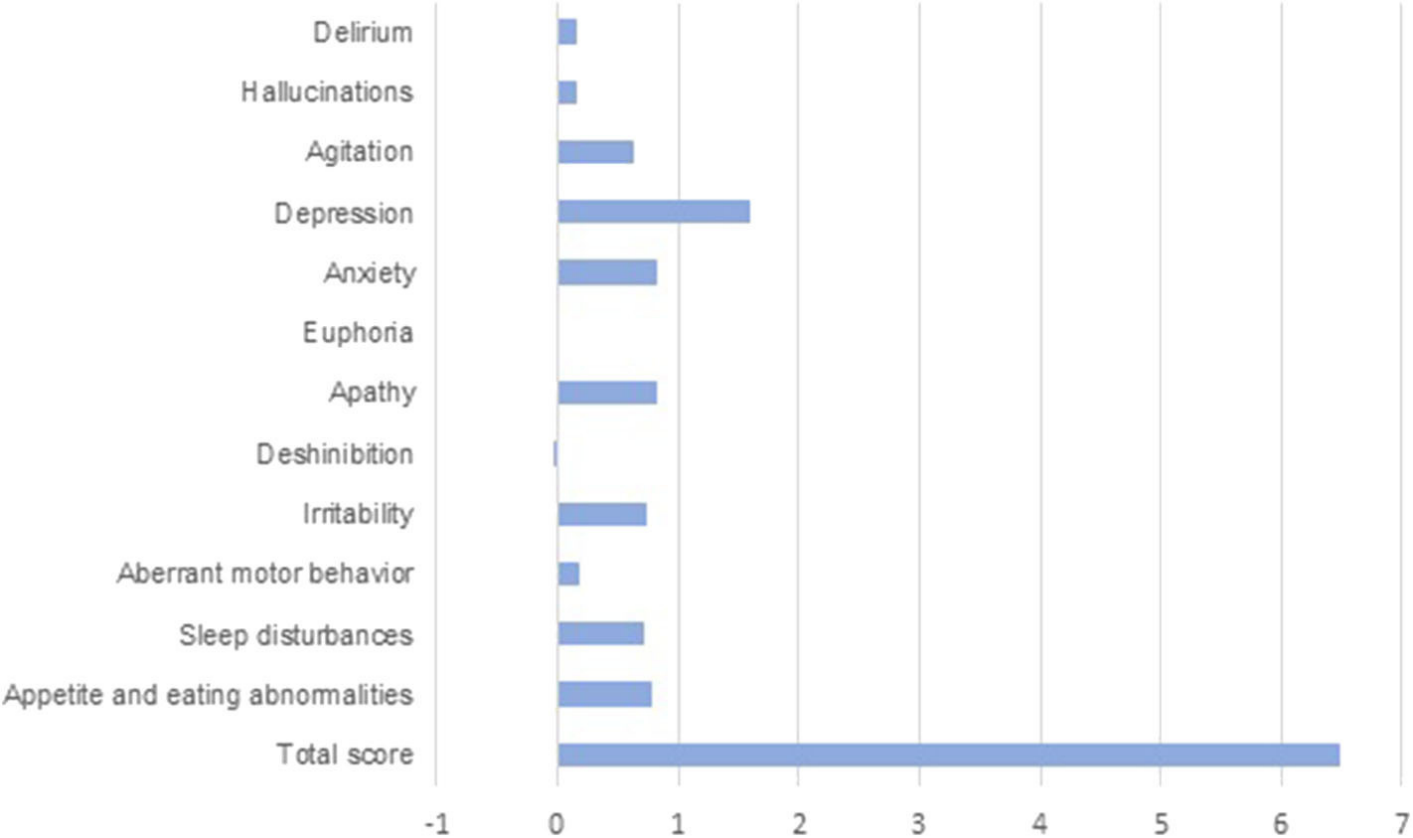
Changes in NPI scores from baseline during lockdown.

Figure 3 shows increases in most of the scores from NPI items during lockdown. An increased score means a worsening in these neuropsychiatric symptoms.

Nine (15%) of the patients presented delirium episodes, 5 of them were patients with severe dementia (CDR 3). 8 (13%) of our patients had increased incidence of falls. Most of the falls (62%) occurred to patients with moderate dementia. No falls occurred to patients with advanced dementia.

Caregivers perceived an increased burden in 25 (41%) cases independently of the dementia stage, though burnout was only reported in 7 (11%) cases, 6, nearly all of them, in cases of moderate-severe dementia. Only 2 (3%) caregivers used support guidelines during lockdown.

When asked about medical care, 10 (16%) of the patients/families interviewed reported difficulties in accessing medical resources. In 20 (33%) cases, medical phone assistance was provided. Only 4 (6%) cases required standard medical care and in 11 cases, nearly 20%, emergency care was needed. 13 (21%) patients had changes in psychopharmacological therapies during lockdown, mostly among the most advanced cases of dementia.

## Discussion

The COVID-19 pandemic has represented a challenge for us all, both health care professionals and patients and caretakers. Our aim in this study was to describe the influence of restrictive measures on patients living with mild cognitive decline and dementia and their caregivers, especially regarding the change in their routines and location, functional performance, neuropsychiatric symptoms, caregiver stress, and to evaluate their perception of sanitary care accessibility during the state of alarm.

Up to 10 (17%) of patients from our sample changed home in order to live with their relatives, which implies a major change for patients living with dementia *per se*. Dementia patients usually suffer from delirium when moving from location (i.e., during weekends or holidays), and usually need an adaptation period, sometimes including pharmacologic treatment adjustment. Twenty percent of our patients lived alone during lockdown which has made it challenging for families to provide optimal care. Although visits to dependent relatives were permitted, the frequency of visits might have been different during the state of alarm.

Regarding SARS-CoV2 infection, surprisingly, all of the patients from our sample who were affected by COVID-19 were also in an advanced stage of dementia. This fact makes us think that there might be a higher transmission due to ineffective isolation or institutionalization. Patients living with severe dementia have more difficulties to maintain complete isolation as they are dependent on basic activities of daily living. On the other hand, there have been multiple outbreaks in nursing homes during the state of alarm. In fact, 3 of our infected patients were institutionalized.

More than half of our patients abandoned previous activity during lockdown (60%). Among all the previously mentioned ceased activities, cognitive stimulation programs in day care centers were discontinued. This may be a step backwards for a lot of patients in early stages benefiting from this stimulation. Some of them also stopped taking care of activities that previously kept them active such as shopping or informal social meetings.

On the other hand, we have to keep in mind that 32% did not experience any change in their daily activities, which implies that there was a previous lack of cognitive and physical stimulation in most of these patients. Thus, we might acknowledge that a substantial amount of our patients is not usually taking either physical or cognitively stimulating activities.

Although no statistically significant changes in CDR were observed, caretaker-perceived cognitive worsening was reported by more than half our respondents (60%). It might be partly due to an increased observation from their caretakers (longer cohabitation) and partially influenced by caregivers' anxiety. An objective evaluation of cognitive status was not included in this assessment due to the limitations of the type of visit and the type of sample (including patients in advanced dementia stages which makes the use of telephonic versions of cognitive scales difficult to perform). We might argue that the cognitive differences sensed before may eventually surface when we get back to normal and therefore unveil as real and daily functional problems. It is important to mention that at the time this paper is written, we still haven't returned to that point of normality when our patients are able to carry their previous lives.

As discussed before, though there might also be cognitive impairment, neuropsychiatric symptoms are clearly aggravated. In our study, we found grounds to believe that the lockdown has significantly worsened this sphere globally, but even more in items such as depression, anxiety, agitation, and loss of appetite. In our view, though this might be influenced by the caregivers' observation, caregivers' own anxiety is a significant element to keep in mind. Furthermore, the fact that our patients developed more affective symptoms than psychotic symptoms during lockdown might reflect the situation of loneliness they have to face. The loss of resources, social gatherings, and institutional follow-up may make them feel forsaken.

Our results are congruent with other current studies such as the one by Lara et al. (11) which have found significant changes in NPI scores, such as agitation, apathy, and motor aberrant activity. The population analyzed in the cited study has a sample with different characteristics, with all patients in stages of mild cognitive impairment and mild dementia. This might explain the difference with our study in which different stages of dementia and etiologies are included.

Other clinical changes have also reported, such as 15% of our patients presented delirium episodes during lockdown. They were all in severe stages of dementia and, therefore, this makes us think that these patients are more fragile and vulnerable to changes. Also, falls rose over 13% in our total sample. Lack of physical stimulation may worsen motor capacities and therefore increase the risk of falls. Our results show that patients with advanced dementia had the least falls of all. This might be related to closer supervision or immobility.

Multiple studies have been conducted over anxiety among the general population in different countries. In Spain, a study by Rodriguez-Rey et al. (16) showed severe psychological impact by the pandemic in 30.4% of their sample. Hence, such psychological impact is present not only in our patients but also in their caregivers.

Our research reveals that 41% of caregivers noticed a subjective increase in stress. We need to keep in mind those figures on the overall population's psychological stress plus their particular situation hereby, taking care of their dependent relative.

In this way, most cases of burnout have been reported in severe dementia stages. This could be possibly related to limitations in outpatient health circuits, such as day-care centers shutdown, consequently reducing any assistance down to their relatives at home only. The fact that most of these challenged caregivers haven't sought help among guidelines and associations (only two of them had) is in most cases because they are not aware that such aids exist at all. That was a fact duly checked throughout our telephone interviews. In order to support these families, it will be necessary to reach out to them with wider promotion campaigns and education on virtual resources.

Medical care has also been affected during lockdown. Our results show that 16% of the families reported real obstacles accessing medical care although telephone assistance was given generally. It is important to remark how soon -within days- on-site attention turned into virtual attention to understand the general confusion of families and patients. Changes in pharmacological therapies reported by the families were made mostly by emergency clinicians and telephone calls. For instance, our data regarding NPI scores changes show higher incidence of anxiety and depression than that of pharmacological changes, which lead us to wonder if this lack of clinical follow-up or availability could have left patients unattended while symptomatic.

In conclusion, although the current uncertainty regarding the evolution of the pandemic makes strategy difficult, the reinforcement and adaptation of the care of these patients will be necessary and urgent. In our study, we exposed the influence of the lockdown in the cognitive, social, and neuropsychiatric spheres of our patients. We need to reach to the caregivers and patients and give them the education and support necessary to deal with the pandemic and social isolation. It is necessary to develop adequate strategies to restore stimulation and activity in our patients in order to improve their and their caregivers' daily life.

A multidisciplinary perspective will for sure be needed, from reinvention of current telemedicine techniques to implementation of preventive long run measures.

### Limitations

All our interviews were made by telephone due to the pandemic situation even after the state of emergency was lifted. This, of course, made the clinical interview occasionally more difficult and could interfere in NPI/CDR values. An objective cognitive evaluation of patients would have been of high interest but due to the type of visit and limitations of telephonic cognitive scales on advanced dementia patients was ruled out. Other pandemic related limitations such as reaching to patients and families also influenced the sample size of our study.

Previous NPI was retrospectively based on medical records and family interviews regarding the previous state of our patients. This is of course less exact than having an actual previous NPI scale. It could magnify the difference between the already present items, for example, if a patient already affected by depression has to stay locked down, the family will magnify this during cohabitation, even if it's his/her usual state.

In some cases, through literature (17), pretest self-assessment has been considered as a valid tool. In our case the NPI retrospective items were asked to a reliable source, such as caretakers and close families.

When patients live in nursing homes or other kinds of institutions, the interview was still made with the families/caregivers. The information we have therefore relies on what the family gets from caretakers and scarce telephone conversations with the patients.

## Data Availability Statement

The raw data supporting the conclusions of this article will be made available by the authors, without undue reservation.

## Ethics Statement

The studies involving human participants were reviewed and approved by Comitè Ètic d' Investigació Clínica del Parc de Salut Mar (CEIM). The patients/participants had provided their signed informed consent to participate in DegMar registry and gived verbal informed consent to participate in this this CogVid study, which was ratified by written consent when possible, as approved by CEIM.

## Author Contributions

Patient recruitment was managed by AB, AF-L, and IE-G. The first draft of the manuscript was written by AB. All authors commented on previous versions of the manuscript, contributed to study conception and design, read, and approved the final manuscript.

## Conflict of Interest

The authors declare that the research was conducted in the absence of any commercial or financial relationships that could be construed as a potential conflict of interest.
