# Supplementary material for: Effects of COVID-19 Pandemic Confinement in Patients With Cognitive Impairment
Source: Front Neurol. 2020 Nov 24;11:589901. doi: 10.3389/fneur.2020.589901 (PMC7732426; doi:10.3389/fneur.2020.589901)
Supplement: Supplementary file 1 [file Annex.pdf]

ANNEX 1 CogVid Hospital del Mar Questionnaire

DEGMAR ID:

Gender

- Male
- Female

Date of birth:

Previous iagnosis

- MCI
- Alzheimer's disease
- Vascular dementia
- Lewy body dementia
- Fronto-temporal dementia
- Mixed/Others

Baseline situation

Social situation

- Institutionalized
- At home
- Supervised at home

CDR:

Previous cognitive stimulation activities

- Yes, in a center
- Yes, exercises at home
- No

During confinement. From March 2020 to May 2020

Have you performed confinement?

- Yes
- No

Have you had difficulty complying with the rules of the alarm state?

- Yes
- No

Have you changed your residence?

- Yes
- No

Have you lived alone?

- Yes
- No

Have you regularly received visits (at least three times a week)?

- Yes
- No

Have you stopped doing activities that you previously did?

- Yes
- No

What activities have you stopped doing?

- Social meetings (walks, playing cards...)
- Daycare center
- Cognitive stimulation in a centre
- Visiting relatives
- Taking care of other family members
- Sport
- Shopping
- Reading
- Watching TV

Have you stopped receiving external social support that you previously received (home assistance, daycare centre...)?

- Yes
- No

Do you think you have had greater difficulty accessing health resources during confinement?

- Yes
- No

Have you received any health care assistance?

- Yes, telephone consultation
- Yes, on-site consultation
- Yes, urgent consultation
- No

Have you declined to consult for fear of possible contagion?

- Yes
- No

In general, have you noticed a worsening in your mood or behavior?

- Yes
- No

In general, have you noticed a worsening of your cognitive state (aspects such as memory, language, attention/concentration...)?

- Yes
- No

Have you suffered any confusional episodes?

- Yes
- No

If so, has it required adjustment for psychotropic drugs during confinement?

- Yes
- No

Previous NPI - Intensity and frequency

NPI during confinement - Intensity and frequency

Have you been ill during the lockdown?

- Yes
- No

Have you had coronavirus?

- Yes
- No

Other infections/respiratory symptoms?

- Yes
- No

Have you required admission or consultation in the emergency room for this reason?

- Yes
    - No Have you required

oxygen therapy?

- Yes
- No

Has anyone close to you gotten sick from COVID-19?

- Yes
- No

Have you had any falls?

- Yes
- No

Have you fallen more often than in previous months?

- Yes
- No

Have you suffered head trauma?

- Yes
- No

Have you had the usual caretaker during confinement?

- Yes
- No
- Not applicable

Have caregivers had a subjective perception of increased burden?

- Yes
- No
- Not applicable

Have caregivers had a subjective perception of burnout or claudication?

- Yes
- No
- Not applicable

Have you used any guidance/advice or support for caregivers during confinement?

- Yes
- No
- I didn’t know this

Post-lockdown situation

Social situation

- Institutionalized
- At home
- Supervised at home
- Deceased

Current CDR:

Cognitive stimulation activities

- Yes, in a center
- Yes, exercises at home
- No
